# Gastric and Intestinal Disorders of the Horse1A paper read before the Graduates’ and Students’ Society, Melbourne Veterinary College.

**Published:** 1901-02

**Authors:** 

**Affiliations:** Government Veterinary Surgeon and Chief Inspector of Cattle, South Australia


THE JOURNAL

OF

COMPARATIVE MEDICINE AND

VETERINARY ARCHIVES.

VOL. XXII. *FEBRUARY, 1901.* NO. 2.

### GASTRIC AND INTESTINAL DISORDERS OF THE HORSE.[1]

BY VETERINARY SURGEON DESMOND,
GOVERNMENT VETERINARY SURGEON AND CHIEF INSPECTOR OF CATTLE,
SOUTH AUSTRALIA.

MR. PRESIDENT AND GENTLEMEN: I feel honored in having the pleasure of reading a paper before this Society, and trust that my observations on the diagnosis and treatment of gastric and intestinal disorders of the horse will cause a discussion which will be the means of making every member of this Society express his ideas as to how this subject should be handled.

In handling such a large subject, and in the last decade of the nineteenth century, when science, especially veterinary science, has made such progress, I think it would be as well to avoid calling every disorder of the horse which arises from affections of the stomach and intestines, colic or gripes. In such cases if a careful physical examination were made, and by one armed with the stethoscope and clinical thermometer, the exact seat of the trouble could be located, which would be a more scientific method than treating with opium to check the pain and allowing nature to remove the cause. Surely an up-to-date veterinarian would not treat impaction of the stomach in the same manner as impaction of the colon? Although we must admit it is pain that kills, still the time has gone by in which veterinarians can keep a practice going with a pound of opium and a curved knife.

We will first deal with acute gastric indigestion, known under the following names—gastric impaction, stomach staggers, gastric plethora, etc.

*Causes.* We will place these under two heads, viz., predisposing and actual. The first (predisposing) are diseases of the organs of digestion, as the teeth and salivary glands; while

[1] A paper read before the Graduates' and Students' Society, Melbourne Veterinary College.

diseases of the liver and spleen may cause reflex action. One of the most important is disease of the stomach itself. The actual causes are : over-gorging is the great cause in most if not in all cases ; rapid feeding, especially when the food is bolted without masticating it properly; errors in dieting, and the water supply. The food which gives rise to these conditions is divided into two heads : 1. Succulent, such as green barley, green clover, green maize, brewers' grains, and growing wheat. Wheat is the worst of all, and I think this applies to both growing wheat and that which is prepared for the mill. 2. Dry, such as seconds, beans, peas, wheat, barley flour, pollard, maize, wheaten straw, and old rye-grass hay, such as is given to cattle by the dairy farmers in the winter to fill out their bellies to allow the rain to run off them. Wheat and some of the green foods are not only blamed for causing indigestion, but may generate a ferment, while some observers go so far as to argue that a poison is formed, while some assert that alcohol in some form is produced and gives rise to intoxication, and in this way they account for the peculiar head symptoms seen in some of these cases.

*Symptoms.* The symptoms in these cases are not well-marked pain. The horse may appear dull, languid, and refuse the most tempting food. As time goes on he begins to show abdominal pain, such as lying down, pawing, yawning, turning up his nose, or, to be more correct, extending his upper lip, and will often extend himself as far as possible, which suggests that he wishes to put his stomach on the stretch—a movement often made by man when attacked by acute indigestion. When lying down the animal will lie on his left side and put his nose back in the region of the stomach on the right side, but not in the flank, as is usually the case in intestinal troubles or derangements. As time goes on he shows more decided symptoms, colicky pains increase, and in some cases pain is shown on pressure over the gastric region. If tympany is associated with gastric impaction—and this is nearly always the case when succulent food has been given—the symptoms are most alarming, as in these cases the animal's breathing is more or less labored owing to pressure on the diaphragm. The pulse in the early stages is usually full, moderately strong, and slightly increased in frequency ; the mucous membranes are slightly injected, while the temperature in the early stages is scarcely more than normal.

If the animal does not obtain relief in the early stages all the symptoms are aggravated ; the pulse increases and becomes irritable; the breathing more labored and difficult; the mucous membranes highly injected, and the temperature may rise to 104° F. In cases where tympany occurs we get eructations of gas, which has that peculiar sour smell well known when the stomach of the horse is opened in post-mortem examinations while the body is warm. When tympany of the stomach is present we may also get tympany of the bowels. In some cases the horse will attempt to vomit, and in a few cases will succeed, the sour, vomited ingesta passing down the nostrils. This condition, usually a fatal symptom of ruptured stomach or strangulation of the first part of the intestines (a splendid specimen of this condition was exhibited to the meeting), may give temporary relief. A few cases are recorded where the horse has vomited and recovered, still the number of cases is so small that the observers have availed themselves of the opportunity to record the cases as something out of the ordinary.

If the above symptoms are continued brain symptoms set in. These are described as dumb staggers, mad staggers, or gastric vertigo. In the dumb form the symptoms are similar to coma; the animal has slight unconsciousness; the head hangs down or is placed in a corner; the pupils are dilated; the gait is unsteady when the animal is made to move; the veins are distended, and if you bleed the blood is thick and dark in color; the mucous membranes are also very dark. The animal may die in a state of coma or convulsions. In the mad form we may get delirium; in fact, the animal "runs amuck." The pulse in this form is rapid, full, and strong; bleeding has little effect on it; the blood flows rapidly, which is not the case in the early stages. The respirations are rapid and the mucous membranes of a bright red color. According to some authorities the bloodvessels of the brain in this form are empty, while others argue that the symptoms are due to congestion of the small bloodvessels of the brain.

When investigating such conditions great care has to be exercised in making post-mortem examinations. The head should be examined first—*i. e.*, before the body is opened—as in opening the trunk first we may drain the blood from the brain and so alter the conditions. If we first open the thorax and abdomen we allow the pressure of the atmosphere to act on the bloodvessels in this region, which may force the blood into the vessels of the brain.

*Diagnosis.* In the early stages it is not an easy matter to diagnose gastric derangement, and especially if tympany is not present; still, if we get the history of the case, examine the food and the surroundings, we may secure some data to guide us in making a correct physical diagnosis. In all cases it is very necessary to examine the patient carefully before medicine in any form is given, as neglect in this respect may lead to fatal results. Diagnosis is difficult when the disorder is accompanied by impaction of the bowels or tympany of the same. I have adopted the following method in making a physical examination in cases where the history, food, and surroundings have led me to believe that impaction of the stomach is the trouble: First, note the appearance of the mucous membranes, then the beat and character of the pulse, or, better still, the action of the heart; also the breathing, noting if it is thoracic or abdominal. While these examinations are in progress the clinical thermometer can be put in the rectum to record the temperature. The next step is to examine the abdominal cavity. This is accomplished in the following manner: Over the posterior third of the sternum the binaural stethoscope is placed, with a view of catching the borborygmus of the anterior portion of the small intestines. If loud peristalsis does not exist the next step is to lift the abdomen with both hands, standing on the left side of the animal, immediately posterior to the sternum. If impaction of the stomach is present and accompanied by pain the animal will turn its head toward this region. Having examined the gastric region, it is necessary to examine the bowels. On the right side behind the last rib, low down in the flank, the stethoscope is to be placed. If tympany exists, loud and quick peristalsis will be heard. If any of those present decide to adopt such a method of examination it would be as well to remember that "practice makes perfect," and this method requires a lot of practice to master the subject; but, with constant practice, one's fingers seem to have eyes in them. Another point of importance to be noted, viz.: it is advisable to be well acquainted with the normal sound of peristalsis in a horse in a normal state.

The method of examining the cæcum and colon will be described when dealing with the derangements of these organs.

One must be very careful in giving an opinion as to the ultimate results in these cases. A prognosis given in the early stages may be regretted. It is better to wait some time before giving a decided opinion, and special care is required with pregnant animals. The after-effects to be dreaded are laminitis, rupture of the stomach or diaphragm, congestion of the lungs, and brain symptoms.

Treatment. This must vary according to the conditions that have caused the derangement, viz., dry or green food. If the troubles have been caused through dry food, and no tympany is present, a full dose of purging medicine combined with nux vomica should be given, followed by hypodermic injections of morphine combined with atropine. In the choice of antispasmodics I do not advise following up a purge with opium or any other sedative *per orum*, and will give my reasons further on in this paper.

If the trouble is due to green food, and tympany is present—a condition which in most cases we have to contend with—the treatment suggested is carbolic acid and camphor combined and given in a solid form. (The carbolic acid acts as a local anæsthetic and the camphor as an antispasmodic. Dose : Two drachms of each.) Other medicines are given, such as sodii bicarb., ammon. aro., and sometimes alcohol. (*As far as my experience reaches, I am of opinion that alcohol in any form is contraindicated.*)

If the above-mentioned drugs have had the desired effect it is advisable to follow up with a physic ball. Hot fomentations kept up for several hours and followed by a stimulating liniment, or an application of ammonia well diluted with water and applied with a blanket, are advised; but in this country it is rare to find owners or attendants with perseverance enough to give the necessary attendance to a method of treatment which, to have the desired effect, must be kept up for several hours.

If the pulse is bounding in character and the animal shows symptoms of brain troubles it is advisable to bleed liberally. If bleeding is resorted to it is now advisable to remove the blood quickly and in quantities that will have a marked effect on the pulse. If the pain still continues try such drugs as chloral hydrate, belladonna, Indian hemp, aconite, or, better still, hypodermic injections of aconitine, morphine combined with atropine. If these remedies above mentioned will not control the pain, or if vomition is attempted, make sure that there has not been an error made in the diagnosis.

*Post-mortem Appearances.* Where one is unfortunate enough to have a death from gastric troubles and has the curiosity and energy to make a post-mortem examination the following conditions are usually found. There are usually three causes : brain troubles, rupture of the diaphragm, or rupture of the stomach. If death has been caused through some affection of the brain (may be toxic) and the examination is made a short time after death (I have often made a post-mortem examination before reflex action of the muscles and peristalsis have ceased) the stomach is usually found intact, but very much engorged with food. The bloodvessels are congested and the blood in them of a dark color. This condition also applies to the bloodvessels of the brain. When the stomach is ruptured it is usual to find the contents scattered among the mesentery and bowels and the mesentery lacerated. The rupture of the stomach usually takes place near the cardiac extremity or along the great curvature. Great care has to be exercised in forming an opinion that rupture of the stomach has been the cause of death. Rupture of the stomach may take place after death. In *ante-mortem* rupture we find laceration with blood in the tissues. If the horse has lived any time after the rupture has taken place we must be guided by the inflammatory condition of the coats of the stomach. If the rupture has taken place after death the edges are clean, pale, and with no inflammatory conditions.

Rupture of the diaphragm may be *ante-mortem* or *post-mortem*. The conditions are much the same as in rupture of the stomach. In *ante-mortem* rupture the lesions are usually near the pillars, while in *post-mortem* rupture the seat is usually the fleshy rim—a condition often seen when death has taken place some time previously and the body is greatly distended with gas.

Affection of the Intestines. This affection is, in my opinion, the most common cause of the ordinary everyday colic or gripes.

*Symptoms.* As in affections of the stomach, the symptoms in the early stages are not those of well-marked pain ; the animal is dull, refuses its food, or eats a little at intervals ; the pulse, respirations, and temperature are not disturbed at this stage. As time goes on the animal shows more or less pain or uneasiness, such as smelling at the bedding and walking around the loose box, or attempts to lie down. In course of time he usually paws, sometimes with the hind legs, and kicks at his belly, puts his nose into the flank, lies down, usually flat on his side, and occasionally brings his head slowly toward his flank with an anxious expression. There may or may not be tympany. If present the symptoms are usually more aggravated. Sweat is found under the flank and in the region of the thighs ; the eyes have an anxious look; reels while walking, labored breathing, and will in many cases throw himself down instead of, as in health, lying down quietly. Another important symptom of impaction of the colon is walking backward, which is usually seen when this affection has lasted some time. The pulse at this stage is increased and the mucous membranes are injected. A very common symptom is frequent micturition, the animal voiding a few drops of urine at a time.

This frequent micturition is, in my opinion, caused by the irritation set up in the colon ; it may be caused through pressure on the bladder or reflex irritation. The frequent micturition leads the owner to believe that the horse has bladder or kidney trouble, often termed "water gripes." How often are we asked to treat a horse in which the owner will say "he is bad in his water, and can make only a few drops at a time." The irritation or pressure causes the animal to micturate as often as a few drops of urine are lodged in the bladder. This can be proved by a physical examination of the bladder *per rectum*.

*Diagnosis.* With derangements of the colon this includes tympany and impaction. The diagnosis is not as difficult as in other portions of the digestive apparatus. First get the history of the case, examine the feces that have passed, the character of the food, and the manner in which it is given ; also the water that is supplied. Next take the pulse, respirations, and the temperature. Note if the pulse is over 60. The next step is to carefully auscultate and palpate the bowels to find if there is normal, increased, or diminished borborygmus going on. This is most important, as I find that in impaction of the colon, on placing the stethoscope on the right side behind the last rib, peristalsis is almost absent. The next step is to explore the rectum. Give an enema of hot water and soap, noting if the injection brings away feces, and if they are hard and coarse or covered with mucus. Soap the hand and arm and place in the rectum ; remove and note appearance of the feces if present, and then ascertain the condition the bladder is in. If the bladder contains urine in any quantity remove it with the catheter. After emptying the bladder again explore the rectum to try and find out the condition of the pelvic flexure of the colon. If the colon is much distended it can with ease be felt through the rectum, to be forced back into the pelvic cavity. If the colon is only slightly distended, peristalsis is diminished, but can be heard all over the abdominal cavity; and if the feces are hard, coarse, and covered with mucus we have, as a rule, simple impaction of the colon. If we have tympany and a metallic sound in the bowels there may be many complications, and one must be careful in giving a diagnosis and prognosis.

TREATMENT. In simple cases of impaction of the colon (I do not call cases that have a pulse over 60 simple cases, and will give my reasons later on in this paper) the first step is to give a physic ball, followed by an intratracheal injection of eserine and pilocarpine. If the eserine and pilocarpine has had the desired effect the animal will be free from pain in from one to two hours. If the eserine treatment cannot be used on account of its expense or through not having a suitable syringe, the next best treatment is cannabis indica, chloral and capsicum, hypodermic injections of morphine, followed by a physic ball. I do not agree in giving opium and aloes together. In giving a drench composed of tincture of opium and a solution of aloes we first lock up the bowels with the opium and then try and unlock them with the aloes. Hypodermic injections of morphine, given in full doses, cause morphine intoxication, in which the horse walks in a circle for six to eight hours. This may lead the owner to imagine it is pain that makes the horse continue walking in a circle.

IMPACTION OF THE RECTUM. I can give the notes of one interesting case of impaction of the rectum—a condition often found in draught stallions that are forced for show purposes and do not get sufficient exercise. While in practice in the Warrnambool district I was summoned by an urgent telegraph message to attend a valuable thoroughbred mare. After travelling fifty miles by rail to see the case the mare was found in the following condition: Pulse, respiration, and temperature normal; prolapse of the rectum, and having the appearance of three knobs, each knob about the size of two clenched fists.

*History.* A week before this mare was found in this condition she was in full training. She was then turned out in a paddock with long and very dry grass. When visited a week after being turned out it was found that there was no water in the paddock. This may have been the cause of the trouble.

*Physical Examination.* Rectum impacted with hard feces; the lumen of the gut was so much indurated that it was found impossible to insert the nozzle of an ordinary Reid's enema pump.

TREATMENT. An emulsion of extract of belladonna and glycerin was passed into the rectum with the aid of a veterinary male catheter, and after using half a gallon of the emulsion about half a barrelful of hard feces were expelled in twelve hours. A purge of pure linseed oil and chloroform was given. The emulsion was continued for three days, when the rectum resumed its normal condition.

The owners of draught stallions and stud grooms have a crude method of dealing with impaction of the rectum. They proceed to remove the hard feces with the half of a small file protected with a cork.

IMPACTION OF THE CÆCUM. With this affection it is not necessary to go into the matter so fully as in other disorders of the intestinal tract. In the diagnosis and treatment the same lines as those suggested for the colon are advised. When the cæcum is very much distended with ingesta it is possible to palpate it by exploration through the rectum.

CONCLUSION. In dealing with gastric and intestinal disorders of the horse the first thing to be aimed at is the location of the trouble—an object which can be accomplished only by a physical examination and a careful consideration of the history. In deciding on a course of treatment the following have to be considered: Eserine sulph. and pilocarpine mur. by intratracheal injection are contraindicated in impaction of the stomach. The alkaloid of calabar bean causes violent contractions, while jaborandi excites the salivary glands and causes a free flow of saliva; when given in excess the saliva cannot be swallowed as fast as it is secreted. As the horse cannot vomit it is dangerous to cause violent contractions of an overloaded stomach, as the contents cannot escape fast enough through the pyloric end to prevent rupture. Intratracheal injections of eserine are far from being satisfactory when the pulse is over 60. When eserine is given and it fails to produce increased peristalsis, passing of flatus and feces, the case is not simple impaction without complications, but inflammatory in its nature. In a paper which I trust to have the opportunity to read before this Society at a future date I will give my notes on the administration of eserine sulphate and pilocarpine mur. by intratracheal injection in over five hundred cases.

The following " dont's " are to be noted in administering eserine: impaction of the stomach, when the pulse is over 60; in pregnant animals with a distended bladder.

*Opium.* I cannot give any notes on the administration of this drug, as I have not used a grain of opium in bowel disorders of the horse during the last ten years.

*Chloral Hydrate.* This is a valuable drug combined with capsicum in impaction of the different portions of the intestinal tract. In disorders of the cæcum and colon it is best given in fluid form—*i. e.*, chloral hydras., six drachms; tincture capsici fort., 1-3, six drachms; water, to one pint.

*Cannabis Indica.* This drug, in my opinion, is the most valuable of the sedatives used in veterinary practice. In stomach troubles it is best to use the extract in the form of a bolus, while with cæcum and colon troubles it is best given in the form of a drench suspended in dilute mucilage. It has the following advantages over opium: it does not cause cerebral excitement and does not lock up the bowels. In full doses it deadens pain and causes sleep, and will put an animal on its food quickly after a shaking up with an intratracheal injection of eserine followed by a physic ball. Its drawbacks are: it is like aconite in not being always of a standard strength—a difficulty that can be gotten over by giving it boldly, as Professor Fred Smith reports that it will not cause death even with enormous doses.

Gentlemen, this subject is like the brook. I must claim your indulgence for reading such a long paper, which I trust will be the cause of a very warm discussion this evening.

